# Circulating miR-23b-3p, miR-30e-3p, and miR-205-5p as Novel Predictive Biomarkers for Ramucirumab–Paclitaxel Therapy Outcomes in Advanced Gastric Cancer

**DOI:** 10.3390/ijms252413498

**Published:** 2024-12-17

**Authors:** Emanuele Piccinno, Annalisa Schirizzi, Viviana Scalavino, Giampiero De Leonardis, Rossella Donghia, Alessia Fantasia, Angela Dalia Ricci, Claudio Lotesoriere, Gianluigi Giannelli, Grazia Serino, Rosalba D’Alessandro

**Affiliations:** 1 Laboratory of Molecular Medicine, National Institute of Gastroenterology, IRCCS “S. de Bellis” Research Hospital, Via Turi 27, 70013 Castellana Grotte, BA, Italy; emanuele.piccinno@irccsdebellis.it (E.P.); viviana.scalavino@irccsdebellis.it (V.S.); 2 Laboratory of Experimental Oncology, National Institute of Gastroenterology, IRCCS “S. de Bellis” Research Hospital, Via Turi 27, 70013 Castellana Grotte, BA, Italy; annalisa.schirizzi@irccsdebellis.it (A.S.); giampiero.deleonardis@irccsdebellis.it (G.D.L.); 3 Data Science Unit, National Institute of Gastroenterology, IRCCS “S. de Bellis” Research Hospital, Via Turi 27, 70013 Castellana Grotte, BA, Italy; rossella.donghia@irccsdebellis.it; 4 Clinical Trial Unit, National Institute of Gastroenterology, IRCCS “S. de Bellis” Research Hospital, Via Turi 27, 70013 Castellana Grotte, BA, Italy; alessia.fantasia@irccsdebellis.it; 5 Medical Oncology Unit, National Institute of Gastroenterology, IRCCS “S. de Bellis” Research Hospital, Via Turi 27, 70013 Castellana Grotte, BA, Italy; angela.ricci@irccsdebellis.it (A.D.R.); claudio.lotesoriere@irccsdebellis.it (C.L.); 6 Scientific Direction, National Institute of Gastroenterology, IRCCS “S. de Bellis” Research Hospital, Via Turi 27, 70013 Castellana Grotte, BA, Italy; gianluigi.giannelli@irccsdebellis.it

**Keywords:** circulating miRNAs, biomarkers, angiogenesis, advanced gastric cancer

## Abstract

Angiogenesis inhibition treatments are limited and are often too late for advanced gastric cancer (GC) patients, in whom its efficacy is reduced. New molecular biomarkers are needed to optimize therapy regimens. In regard to this framework, circulating miRNAs, with high sensitivity and specificity, could be useful biomarkers of GC. The present longitudinal study was focused on analyzing the expression levels of a blood miRNA signature in a cohort of 40 patients receiving second-line therapy combining Ramucirumab and Paclitaxel, stratified based on their Progression-Free Survival (PFS). Using differential and bioinformatic analysis, miR-205-5p, miR-30e-3p, and miR-23b-3p were selected as possible predictive biomarkers, with the results showing that they were more highly expressed in patients exhibiting longer PFS and that they were involved in modulating angiogenesis. Furthermore, patients with longer PFS showed a progressive and significant decrease in the selected miRNA to minimal levels. The loss of the protective effect and the increased expression of the hypothetical targets, including angiopoietin-2, were then observed. The hypothesis was supported by the inverse correlation found for miR-205-5p and angiopoietin-2. Circulating levels of miR-205-5p were protective (HR = 0.37, *p* = 0.02) and patients with higher baseline miRNA levels had longer OS (12.47 vs. 9.00 months). Our findings suggest that these three miRNAs may be novel candidates as non-invasive predictive markers of therapy outcomes.

## 1. Introduction

Gastric cancer (GC) is the fifth most common type of tumor in people over 70 and the fifth leading cause of death from cancer in the general population [1]. In Western countries, 80% of patients are diagnosed with advanced non-resectable disease or relapse within 5 years of having surgery with curative intent. The prognosis for advanced disease remains unfavorable, with a 5-year survival rate < 30% for all stages and <4% for metastatic disease [2,3].

The search for predictive/prognostic biomarkers is crucial for the treatment of locally advanced and metastatic GC. Despite the advent of immunotherapy and new molecular targeted therapies, therapeutic options for these patients remain limited [4,5,6,7]. Patients in fit clinical condition and with adequate organ function are possible candidates for chemotherapy, the gold standard treatment for advanced GC, allowing a median survival rate of about one year compared to 3–4 months for supportive care [8].

The combination of Paclitaxel (PTX) and Ramucirumab is the second-line therapy of choice for the treatment of advanced GC [9,10]. Combination therapy appears to be a promising option to prevent PTX resistance, even in patients with recurrent and metastatic gastric cancer undergoing first-line taxane-based chemotherapy. Nevertheless, no biomarkers are currently available to predict the outcome of anti-angiogenic therapy in GC patients.

Tumor angiogenesis plays a pivotal role in the growth and dissemination of cancer cells. Anti-angiogenic therapy is currently one of the most promising treatment options in clinical oncology, but the response to anti-angiogenic therapy in GC patients remains poor. The increased activity of pro-angiogenic factors alone is not enough to explain tumor angiogenesis, since it is regulated by both activators and inhibitors. Therefore, the identification of inhibitors and biomarkers to predict the response to anti-angiogenic treatment is crucial to improve the outcome for patients with advanced GC [11,12]. In this context, there is considerable evidence that circulating miRNAs could be useful as biomarkers for the diagnosis and prognosis of cancer [13,14,15], since many circulating miRNAs, with high specificity and sensitivity, have been identified in the initial stages of cancer [13]. Moreover, deregulated miRNAs could predict both the response to therapy and disease recurrence. Indeed, in breast cancer, high levels of miR-125b were associated with chemotherapeutic resistance to 5-fluorouracil (5-FU), epirubicin, and cyclophosphamide [16]. In metastatic colorectal cancer, a signature of four miRNAs was correlated with the response to FOLFIRI plus aflibercept treatment [17].

The present longitudinal retrospective study aimed to analyze the expression levels of a signature of circulating miRNAs in blood samples derived from patients with advanced GC undergoing second-line therapy with PTX and Ramucirumab. The goal of this work was to define a group of miRNAs that are involved in the response to therapy and then to correlate their expression with the clinical outcome.

## 2. Results

### 2.1. Clinical–Pathological Features of the Patient Cohort

The clinical data of the patient group examined in this retrospective study are reported in Table 1. The total number of patients in the cohort (*n* = 40) was subdivided into two groups, according to the type of response to the second-line therapy with Ramucirumab and PTX. A clinical and instrumental evaluation (CT) was performed on a quarterly basis, according to the criteria for evaluating the response in solid tumors 1.1 (RECIST 1.1). The group of rapidly progressing patients (RP group) included patients who showed signs of disease progression during the first assessment (PFS ≤ 3; *n* = 18). The controlled disease (CD) group (*n* = 22) included patients with a stable or partial response to the first clinical–radiological control (PFS > 3), who continued with the therapy until there was evidence of progression or therapy toxicity. For each of the two groups of patients, their gender, age, histological type (according to Lauren’s classification), lymph node involvement (N0 vs. N+), and metastasis (M0 vs. M+), as reported at the time of diagnosis, are shown in Table 1. A median Progression-Free Survival (PFS) rate of 2.68 months and a median overall survival (OS) rate of 6.30 months was reported for the RP patients, while the CD patients had a median PFS rate of 10.38 months and 12.47 months for their OS. The Kaplan–Meier curves in Figure S1 show the differences in survival between the two groups of patients.

### 2.2. Identification of Differentially Expressed miRNAs Before Therapy

To identify differentially expressed miRNAs, array profiling of plasma miRNAs was performed at the baseline, before therapy, in a cohort of 22 patients with stable disease, denominated as controlled disease (CD), and in 18 patients with rapid progression (RP) of disease. Among 179 human miRNAs represented in the array, a mean of 154 miRNAs were expressed in each sample. Applying a fold change threshold of 1.5 and a *p*-value of 0.05, we found that 13 miRNAs were differentially expressed between the RP patients compared to the CD patients before therapy (Table 2).

**Figure 1:**
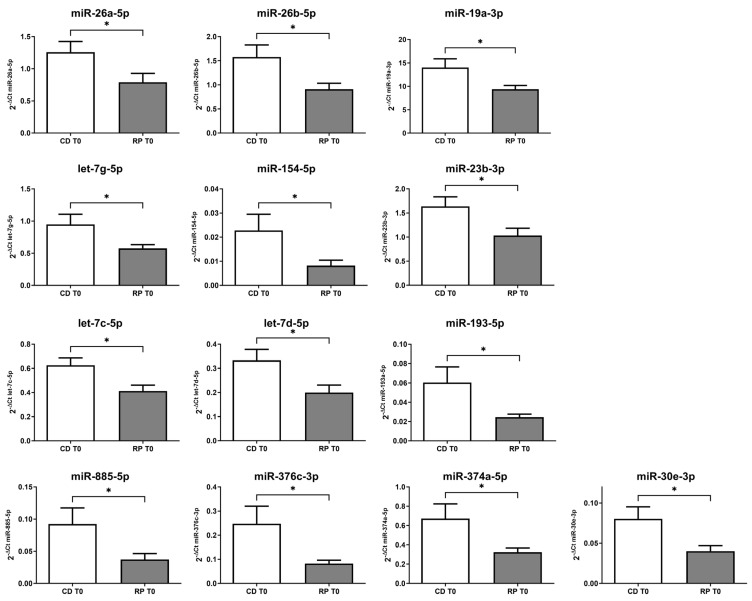
The relative expression using the 2^−ΔCt^ method for differentially expressed miRNAs in the rapid progression (RP, *n* = 18) group versus the controlled disease (CD, *n* = 22) group at the baseline. * *p* < 0.05.

Interestingly, 13 miRNAs were found to be downregulated in the RP patients compared to the CD patients at the baseline, suggesting that these miRNAs could predict the response to therapy. The trends of the selected miRNAs associated with the RP and CD patients are shown in Figure 1.

### 2.3. Analysis of the miRNA Targets and Pathways

To investigate the molecular mechanisms regulated by the miRNAs, we performed bioinformatics analysis to identify the target genes and pathways of the 13 downregulated miRNAs. The pathway analysis, using miRPath and miRSystem tools, revealed that some deregulated miRNAs were involved in the modulation of genes in cancer-related pathways. Indeed, the top relevant pathways selected according to the *p*-value were the TGF-beta signaling pathway, glycosaminoglycan biosynthesis/chondroitin sulfate, the mTOR signaling pathway, focal adhesion, the ErbB signaling pathway, notable pathways in cancer, the Wnt signaling pathway, the PI3K–Akt signaling pathway, and the p53 signaling pathway (Table S1). Notably, “pathways in cancer” signaling included genes involved in angiogenesis, specifically the VEGF signaling pathway.

In previous work, we identified three angiogenic biomarkers that were able to predict the response to treatment with Ramucirumab and PTX, namely *VEGFC*, VEGFR3 (*FLT4* gene), and ANG-2 (*ANGPT2* gene) [13]. Here, we evaluated the putative binding sites in these three genes for the 13 identified miRNAs. We found that most of these miRNAs could regulate *ANGPT2*, *VEGFC,* and *FLT4* genes (Table 3 and Table S2).

### 2.4. Identification of miRNAs for Long-Term Responses

In this analysis, we divided the CD patients into two subgroups: patients with a PFS ≥ 8 (*n* = 14) and patients with a PFS > 10 (*n* = 7), comparing each group with the RP patients. In the first analysis, we identified 14 differentially expressed miRNAs between the two groups (FC > 1.5 and *p*-value < 0.05; Table 4). Notably, eight of them were common to the list derived from the comparison with all the CD patients (hsa-miR-26a-5p, hsa-miR-26b-5p, hsa-miR-23b-3p, hsa-let-7d-5p, hsa-miR-193a-5p, hsa-miR-885-5p, hsa-miR-376c-3p, and hsa-miR-30e-3p).

In the second analysis, the comparison between the CD patients with a PFS > 10 and the RP patients underlined 16 differentially expressed miRNAs (FC > 1.5 and *p*-value < 0.05; Table 5). Five of them were common to the list derived from the comparison with all the CD patients (hsa-miR-26a-5p, hsa-miR-26b-5p, hsa-miR-885-5p, hsa-miR-376c-3p, and hsa-miR-30e-3p). Moreover, four newly identified miRNAs (hsa-miR-485-3p, hsa-miR-132-3p, hsa-miR-205-5p, and hsa-miR-378a-3p) were in the two lists, but showed a remarkable difference in terms of the FC.

### 2.5. Validation of Deregulated miRNAs During Follow-Up

**Figure 2:**
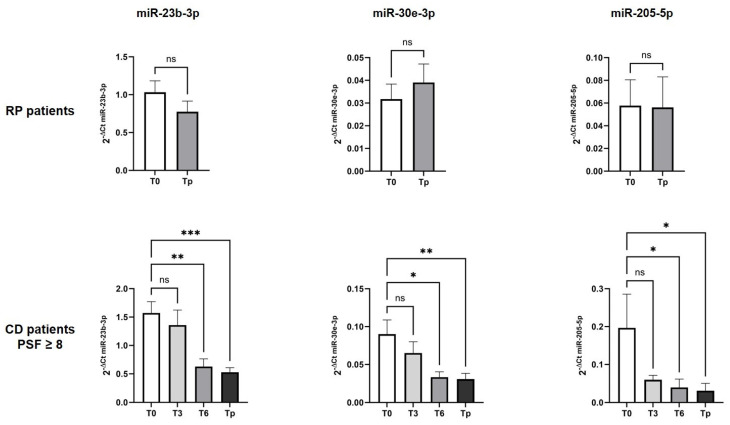
Change in the expression levels of selected miRNAs during therapy (T0, T3, T6, T9, and Tp) in rapid progression (RP, *n* = 18) and controlled disease (CD) with PFS *≥* 8 patients (*n* = 14). The relative expression of miR-23b-3p, miR-30e-3p, and miR-205-5p was calculated using the 2^−ΔCt^ method, reported for each time from the basal level T0 until the disease progression Tp; * *p* < 0.05, ** *p* < 0.01, *** *p* < 0.001, ns: not significant.

In order to evaluate the change in the expression of the miRNAs during the follow-up for the RP and CD patients, we selected eight miRNAs from the three lists based on their involvement in cancer-related pathways and, in particular, in the angiogenesis process (hsa-miR-193a-5p, hsa-miR-205-5p, hsa-miR-885-5p, hsa-miR-26a-5p, hsa-miR-485-3p, hsa-miR-378a-3p, hsa-miR-30e-3p, and hsa-miR-23b-3p). Subsequently, we established the expression of these eight miRNAs for each follow-up timepoint (T3, T6, T9, and Tp), considering the RP patients and CD patients, and then the CD patients with a PFS *≥* 8. Three miRNAs (hsa-miR-23b-3p, hsa-miR-30e-3p, and hsa-miR-205-5p) were found to have a significantly decreased level of expression from T0 to Tp (Anova *p*-value < 0.05; Figure 2) in the CD patients, but not in the RP patients.

This variation was also observed in the CD patients with a long-term response (PFS > 10), in which an additional time point was analyzed (*p* < 0.05, Table 6).

### 2.6. Prognostic Value of Circulating miR-205-5p

**Figure 3:**
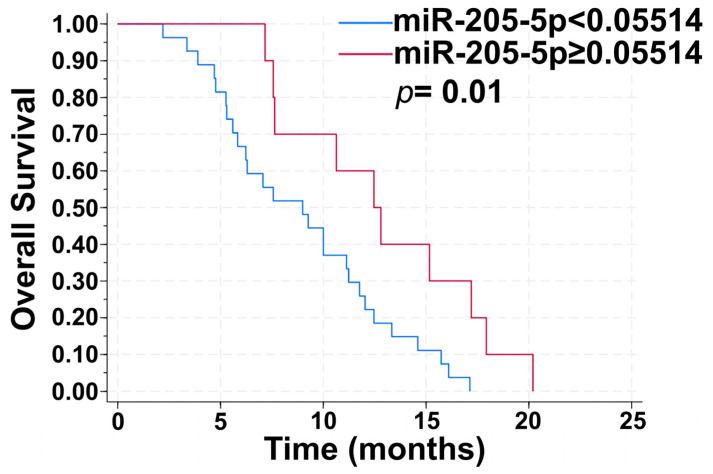
Prognostic value of miR-205-5p. Kaplan–Meier overall survival (OS) plot. A cut-off point for miR-205-5p was fixed by the ROC curve stratification of patients based on PFS ≤ 3 and PFS ≥ 8 (log rank test *p*-value: 0.01).

The Kaplan–Meier overall survival (OS) calculation demonstrated that the basal levels of the selected miRNAs (miR-23b-3p, miR-30e-3p, and miR-205-5p) were associated with the patients’ overall survival. A cut-off point for each miRNA was fixed by the ROC curve stratification of the patients based on a PFS ≤ 3 and a PFS ≥ 8, to analyze the two cohorts of patients based on their response to anti-angiogenic therapy. Patients with miR-205-5p expression levels ≥ 0.05514 had an improved OS rate compared to patients with miR-205-5p expression levels < 0.05514 (12.47 months vs. 9.00 months, *p* = 0.01 CI 95%), showing that miR-205-5p expression levels ≥ 0.05514 had a protective effect (HR = 0.37, *p* = 0.02, 0.16 to 0.86 95% CI) (Figure 3).

### 2.7. Circulating Levels of miR-30e-3p and miR-205-5p Were Correlated with Serum Ang2

**Figure 4:**
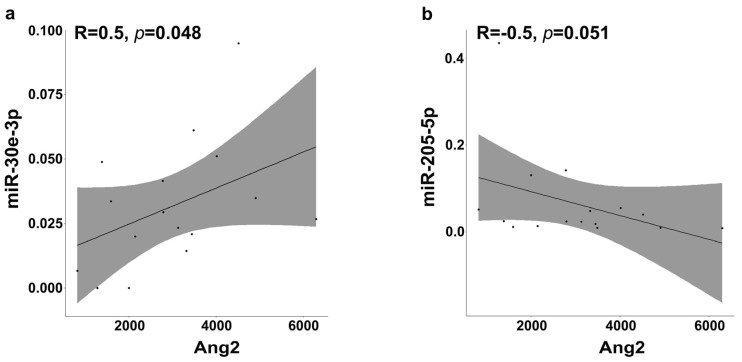
Correlation matrix between circulating Ang2 levels and miRNAs. Spearman’s Rho correlation between serum Ang2 and miR-30e-3p (**a**) and miR-205-5p (**b**) in the total patient cohort.

Correlations between the basal levels of the three selected miRNAs and those of markers involved in angiogenesis [18], and which were previously analyzed in the same patient cohort, including Ang2, VEGFC, and VEGFR3, were investigated. Spearman’s Rho analysis revealed that both miR-30e-3p and miR-205-5p were correlated with serum levels of Ang2 in the total patient cohort. The correlation indexes (ρ) indicated a moderate (R = 0.5) and significant correlation in both cases, directly for miR-30e-3p and indirectly for miR-205-5p (Figure 4).

These results suggest that miR-205-5p could directly target Ang2 and, on the contrary, miR-30e-3p and Ang2 could be related to a feedback loop mechanism.

## 3. Discussion

In the last few years, a growing body of evidence has opened up new frontiers in the treatment of advanced GC, combining traditional drugs with new molecules that are able to counteract tumor dissemination and progression, such as anti-angiogenic drugs. However, anti-angiogenic treatments may have severe side effects [19]. Approved anti-angiogenic agents are currently limited and, in some cases, these drugs are administered too late to be efficacious in regard to the treatment of advanced cancer patients [20]. Both of these factors restrict the design of combination therapy. Thus, there is an urgent clinical need to identify new molecular biomarkers to optimize therapeutic schedules.

MiRNAs, small non-coding single-stranded RNAs, form base pairs with complementary nucleotide sequences of target mRNAs, inhibiting the mRNA and, thereby, regulating gene expression at the post-transcriptional level [21]. Recent studies have identified associations between miRNA expression and tumor growth and progression. Several studies have reported that circulating miRNAs may potentially be used as novel biomarkers in different types of cancer, including gastric cancer [13,22]. The high stability of circulating miRNAs after prolonged incubation at room temperature and/or multiple freeze–thaw processes is well-known. In addition to this high stability, the characteristics of miRNAs, such as tissue-specific miRNA signatures and the availability of many copies per cell, indicate their potential advantages as biomarkers compared to other nucleic acids, such as circulating DNA and mRNA [22].

In the present study, starting from differential expression analysis of circulating miRNAs, we identified a set of miRNAs that might be able to predict the therapy outcome for patients receiving second-line anti-angiogenic treatment (PTX and Ramucirumab). At the baseline, before therapy, we found that a group of 13 circulating miRNAs discriminate between patients with rapid disease progression within the first three months of treatment from patients with a partial response and stable disease. These miRNAs were downregulated in patients undergoing rapid progression, suggesting their possible involvement in tumor progression, as well as their role in predicting early responses to therapy.

Bioinformatics analysis was used to identify the possible target genes and pathways regulated by the identified miRNAs. Pathway analysis revealed that the deregulated miRNAs were involved in the regulation of genes in cancer-related pathways. In addition, among the most relevant pathways, were those involved in regulatory signaling for tumor microenvironment remodeling and angiogenesis.

MiRNAs could act as pro-angiogenic factors triggering angiogenesis and the growth of tumor cells or could have anti-angiogenic properties, inhibiting the expression of key factors in tumor angiogenesis [23,24]. Specifically, we highlighted, among the putative targets of the most differentially expressed miRNAs, the genes involved in the VEGF pathway, such as VEGFC, VEGFR3, and Ang2 [18]. We previously demonstrated that, in the same patient cohort, at the beginning of the third cycle of therapy, the lowering of VEGFC and Ang2 circulating levels was correlated with a lower risk of disease progression and, thus, longer PFS [18].

The analysis comparing the patients with rapidly progressing disease (PFS ≤ 3 months) with the long-term response (PFS ≥ 8 or PFS > 10 months) disease samples revealed differentially expressed miRNAs that were not detected in the initial analysis, suggesting that these miRNAs were modulated by anti-angiogenic therapy.

To assess variations in the expression of the miRNAs during therapy and follow-up, we selected eight miRNAs from the three comparisons, based on their involvement in cancer-related pathways and, specifically, in the angiogenesis process. The expression levels were investigated at the beginning of therapy and after three months of therapy, in both patient groups. In the group with longer PFS, subsequent measurements were considered until disease progression. Among the eight selected miRNAs, miR-23b-3p, miR-30e-3p, and miR-205-5p showed significant differences in their expression from the baseline to the time of progression and the significance was further increased in patients with PFS > 10 months. However, considering the same time points, in the group of RP patients, no miRNA variations were revealed, suggesting their possible predictive role in regard to therapy responsiveness.

Of particular interest is the progressive decrease in the circulating levels of the three miRNAs to a minimum level detected at the time of progression. This finding could be explained by assuming that these miRNAs play a protective role in patients, with a better response to therapy when they are expressed at higher levels. During therapy, the activation of resistance mechanisms could explain the progressive decrease in the expression levels of these miRNAs until they reach minimal values at the time of progression, causing the loss of the protective effect and the increased expression of the target genes, including those involved in angiogenesis. In many studies reported in the literature, an onco-suppressive role is attributed to these miRNAs in regard to several cancers [25,26,27,28,29,30].

A relevant issue that confirms our hypothesis is that circulating levels of miR-205-5p resulted in a protective effect (HR = 0.37, *p* = 0.02, 0.16 to 0.86 95% CI) and the median OS (12.47 months vs. 9.00 months, *p* = 0.01 CI 95%) of patients with expression levels exceeding the cut-off value was significantly higher than in those with lower levels. Furthermore, the basal expression levels of miR-205-5p were found to be inversely correlated with basal levels of Ang2. This inverse correlation could be explained by the presence of putative binding sites of miR-205-5p in the promoter region of the Ang2 gene. Our previous study showed a greater decrease in Ang2 throughout therapy in patients with longer PFS, followed by a significant increase at progression, which could be explained by the progressive decrease in circulating levels of miR-205-5p in this class of patients [18]. Instead, a direct moderate correlation was found between the basal levels of miR-30e-3p and Ang2. Although bioinformatic analysis detected binding sites for this miRNA in the Ang2 gene, this type of correlation could be explained due to the regulation of the miRNA by Ang2.

MiR-205 is known to be involved in multiple physiological, oncogenic, and tumor suppressor pathways [29]. Its expression is regulated by different mechanisms depending on the cell and tumor types, thus contributing to its involvement in many cellular processes. Many factors in the tumor microenvironment, such as hypoxia and the presence of inflammatory cytokines, contribute to miR-205 transcriptional dysregulation [29]. It is well-known that the same miRNA can play an oncogenic [30,31] or onco-suppressive role depending on the tumor context. Growing evidence suggests that miR-205-5p inhibits angiogenesis in several cancers [32,33,34]. Zhang and colleagues, based on data generated from both GC-TGCA and in-house GC datasets, associated reduced miRNA expression in tumor tissues with a worse prognosis. Their suggestion is that the malignant phenotype is caused by increased markers of angiogenesis, VEGFA, and FGF1, resulting from a decrease in the miRNA expression level, indicating a possible clinical application of this miRNA as an anti-angiogenic therapy [32]. We hypothesized that miR-205-5p may play a protective role in GC patients receiving anti-angiogenic therapy. The decrease of miR-205-5p during treatment may contribute, through increased Ang2 levels, to the activation of alternative angiogenic pathways to VEGFR2/VEGFA, such as the Tie2/Ang2 and VEGFR3/VEGFC pathways. Future studies are needed to biologically validate the effect of this miRNA on resistance to anti-angiogenic therapy.

## 4. Materials and Methods

### 4.1. Study Design, Plasma Sample Collection, and Analysis

Plasma samples were obtained from 40 patients, with metastatic GC patients receiving second-line therapy with Ramucirumab and PTX. In this longitudinal analysis, plasma samples collected before the patients started therapy and at the first infusion of each cycle were considered. The study was approved by the relevant Ethics Committee (prot. N°139/c.e. 28 June 2017). Patients provided written informed consent to the study of their blood samples. The miRNA analysis was performed on plasma samples corresponding to the basal level (T0), the third cycle of therapy (T3), and the time of radiological and clinical disease progression (Tp). In the case of particularly strong responses to therapy, analyses were performed at intermediate times during the periodic radiological re-evaluations (T6, T9, T12, etc.). Two different groups of patients were identified, based on the clinical evaluation after three months of treatment: patients who presented with RP and patients who presented with CD with a partial response or stable disease.

### 4.2. Plasma Preparation

Whole blood was collected into commercially available anticoagulant-treated tubes e.g., EDTA treated. Cells, including platelets, were removed from the plasma by centrifugation for 15 min at 2000× *g*, using a refrigerated centrifuge. Following centrifugation, the plasma was immediately transferred into clean microcentrifuge tubes. The samples were aliquoted to avoid multiple freeze–thaw cycles and stored at −80 °C.

### 4.3. miRNA Isolation

The isolation of the total RNA, including the miRNA, was performed from the plasma by means of a Serum/Plasma Advanced Kit, according to the manufacturer’s instructions (Qiagen, Hilden, Germany). The plasma was thawed on ice and centrifuged at 3000× *g* for 5 min in a 4 °C microcentrifuge. One aliquot of plasma was mixed with RPL buffer, vortexed, and left at room temperature for 3 min to ensure complete lysis, and to release and stabilize the RNA from the plasma proteins and extracellular vesicles, and to allow complete inactivation before adding spike-ins (Qiagen, Hilden, Germany). For quality control in terms of sample-to-sample variations during the RNA isolation, a synthetic set of spike-ins, UniSp2, UniSp4, and UniSp5, was added to the RPL buffer and was applied to each serum sample, according to the manufacturer’s instructions. The RPP buffer was used to precipitate the proteins and inhibitors and then isopropanol was added to facilitate the binding of the RNA molecules on the silica membranes. The sample was transferred into an RNeasy UCP MinElute spin column and the total RNA, including small RNA fractions, was eluted using 50 µL of RNase-free water.

### 4.4. Reverse Transcription

The cDNA was synthesized using the miRCURY LNA Reverse Transcription Kit (Qiagen, Hilden, Germany), according to the guidelines provided by the manufacturer, including the spike-in controls. UniSp6 containing *C. elegans* cel-miR-39-3p was added as positive control to each reaction to monitor the cDNA synthesis efficiency. The reaction mixtures were incubated at 42 °C for 60 min and at 95 °C for 5 min. The cDNA specimens were stored at −20 °C until PCR analysis.

### 4.5. miRNA Profiling Using a RT Array

This study was divided into two phases: phase I, miRNA profiling using an array; and phase II, validation using a quantitative real-time PCR.

The expression of the miRNAs was determined in 40 patients using the miRCURY LNA miRNA Serum/Plasma Focus PCR (#YAHS-106Y, Qiagen, Hilden, Germany), which revealed 179 common human serum/plasma miRNAs. Ten µL per well of a premix, including undiluted cDNA template, 2X miRCURY SYBR Green PCR Master Mix, and RNase-free water, was dispensed into the miRCURY LNA miRNA Serum/Plasma Focus PCR system.

The real-time PCR was performed using the CFX96 System (BioRad Laboratories, Hercules, CA, USA) at 95 °C for 2 min, followed by 40 amplification cycles at 95 °C for 10 s and at 56 °C for 1 min. Moreover, miR-93-5p and miR-425-5p were used as endogenous controls to perform data normalization. The relative expression of the miRNAs was calculated using the 2^−ΔCt^ method. For each miRNA, the ΔCt value was calculated by subtracting the geometric mean of the miR-93-5p and miR-425-5p Ct values from the Ct value of each target miRNA.

### 4.6. miRNA Validation Using a Real-Time PCR

The real-time PCR for the quantification of a subset of 8 miRNAs was performed using the miRCURY SYBR Green PCR Master Mix and the miRCURY LNA miRNA PCR Assay for miR-193a-5p (#YP00204665), miR-205-5p (#YP00204487), miR-885-5p (#YP00204473), miR-26a-5p (#YP00206023), miR-485-3p (#YP00206055), miR-378a-3p (#YP00205946), miR-30e-3p (#YP00204410), and miR-23b-3p (#YP02119314), plus miR-425-5p (#YP00204337) and miR-93-5p (#YP00204715), as endogenous controls for normalization (Qiagen, Hilden, Germany), according to the manufacturer’s recommendations. The reactions were carried out according to SYBR Green chemistry, using the CFX96 System (BioRad Laboratories, Hercules, CA, USA). A comparative real-time PCR was performed in triplicate, including no-template controls. The relative expression of specific miRNAs was calculated using the 2^−ΔCt^ method. For each miRNA, the ΔCt value was calculated by subtracting the geometric mean of the miR-93-5p and miR-425-5p Ct values from the Ct value of each target miRNA.

### 4.7. Bioinformatic and Statistical Analyses

The putative targets of the miRNAs were predicted using the miRWalk 3.0 (Heidelberg, Germany) (http://mirwalk.umm.uni-heidelberg.de/, accessed on 21 November 2023), TargetScan 7.1 (http://www.targetscan.org/vert_80/, accessed on 21 November 2023), TarBase v.8 (https://dianalab.e-ce.uth.gr/html/diana/web/index.php?r=tarbasev8/index; accessed on 21 November 2023), RNAcentral (https://rnacentral.org/, accessed on 13 December 2023), miRabel (http://bioinfo.univ-rouen.fr/mirabel/; accessed on 13 December 2023), and miRDB (http://www.mirdb.org/, accessed on 13 December 2023) algorithms. Potential targets of interest were considered by overlapping the results from the six algorithms and selecting the targets of genes predicted by at least two of the algorithms.

Pathway analysis was performed using the miRPath v.3 (https://dianalab.e-ce.uth.gr/html/mirpathv3/index.php?r=mirpath, accessed on 15 January 2024) and miRSystem (http://mirsystem.cgm.ntu.edu.tw/, accessed on 15 January 2024) tools.

The patients’ characteristics are presented as the median and interquartile range (IQR) for continuous variables and as the frequency and percentage (%) for categorical variables.

To test the association between the independent groups (rapid progression vs controlled disease), the Chi-square or Fisher test were used for the categorical variables where necessary, while the Wilcoxon rank sum and Mann–Whitney test were used for the continuous variables, also in regard to the comparison between the miRNA variations (delta) between the groups.

The Wilcoxon matched-pair signed-rank or Friedman test was applied for the continuous parameters to evaluate the variations in the miRNA concentrations at different times. A cut-off point in terms of the miRNAs was calculated (response of the CD ≥ 8.0 vs. RP ≤ 3.0 group), based on the maximized Youden’s index and to minimize the distance to the point (0, 1) in the ROC space.

Survival probability was explored using the non-parametric Kaplan–Meier method, as well as the equality of survival among the groups and miR-205-5p categories, and the log-rank test was used to evaluate the differences between the curves.

The association of miR-205-5p with mortality risk was evaluated using the Cox proportional hazards regression model and the estimate was expressed as the hazard ratio (HR) and the relative 95% confidence interval.

The Spearman rank correlation coefficient was used to test the strength and direction of the associations between the two variables examined.

Graphs were built using GraphPad Prism software version 9.0.0 and expressed as the mean ± SEM. Differences among the experimental conditions were considered statistically significant at *p* < 0.05.

The analyses were conducted using StataCorp 2023 Stata Statistical Software: Release 18 (College Station, TX, USA: StataCorp LLC.), while RStudio (“Chocolate Cosmos” Release) was used for the plots.

## 5. Conclusions

In conclusion, in this study, for the first time, we identified miR-23b-3p, miR-30e-3p, and miR-205-5p as circulating biomarkers that can predict the response to second-line therapy with Paclitaxel and Ramucirumab in advanced GC patients. Our findings suggest that these three miRNAs could be used as novel non-invasive predictive markers to test early responsiveness to therapy.

## Figures and Tables

**Table 1:** Comparison of clinical data between the patient groups (CD vs. RP).

Parameters *	Group		*p* ^^^
	CD (*n* = 22)	RP (*n* = 18)	
Gender (M) (%)	16 (72.73)	13 (72.22)	0.97 ^¥^
Age (yrs)	66.83 (13.78)	68.57 (12.76)	0.83
Histological type (%)			0.73 ^¥^
Intestinal	7 (31.82)	6 (40.00)	
Diffused/mixed	15 (68.18)	9 (60.00)	
T stage (%)			0.67 ^¥^
T0	3 (15.00)	4 (25.00)	
T+	17 (85.00)	12 (75.00)	
N stage (%)			0.35 ^¥^
N0	4 (20.00)	1 (6.25)	
N+	16 (80.00)	15 (93.75)	
M stage (%)			0.006 ^¥^
Mx	1 (5.00)	7 (43.75)	
M0	13 (65.00)	3 (18.75)	
M1	6 (30.00)	6 (37.50)	
PFS II line (months)	8.21 (4.93)	2.68 (1.25)	<0.0001
Median survival (months)	11.77 (6.47)	6.30 (5.23)	0.002

* The median and interquartile range (IQR) for continuous variables and the frequency and percentage (%) for categorical variables. ^ Wilcoxon rank sum test; ^¥^ Chi-square, or Fisher test, when necessary. Abbreviations: CD, control disease; RP, rapid progression; PFS, progression-free survival.

**Table 2:** Differentially expressed miRNA in RP patients versus CD patients. Fold change (FC) in the rapid progression group (*n* = 18) versus the controlled disease group (*n* = 22). The statistical significance of the data was examined using two-tailed Student’s *t* test (*p*-value).

ID miRNA	FC	*p*-Value
hsa-miR-26a-5p	−1.592	0.037
hsa-miR-26b-5p	−1.733	0.029
hsa-miR-19a-3p	−1.500	0.041
hsa-let-7g-5p	−1.646	0.040
hsa-miR-154-5p	−2.785	0.031
hsa-miR-23b-3p	−1.585	0.021
hsa-let-7d-5p	−1.667	0.025
hsa-let-7c-5p	−1.515	0.035
hsa-miR-193a-5p	−2.451	0.032
hsa-miR-885-5p	−2.480	0.043
hsa-miR-376c-3p	−3.020	0.045
hsa-miR-374a-5p	−2.099	0.049
hsa-miR-30e-3p	−2.004	0.025

**Table 3:** Target analysis of putative binding sites of differentially expressed miRNAs in the 3′UTR of the angiogenic biomarkers *VEGFC*, VEGFR3 (*FLT4* gene), and ANG-2 (*ANGPT2* gene).

	*VEGFC*	*FLT4* (VEGFR3)	*ANGPT2* (ANG-2)
**hsa-miR-26a-5p**	✓	✓	✓
**hsa-miR-26b-5p**	-	✓	✓
**hsa-miR-19a-3p**	-	-	✓
**hsa-let-7g-5p**	-	-	✓
**hsa-miR-154-5p**	✓	-	-
**hsa-miR-23b-3p**	✓	✓	✓
**hsa-let-7d-5p**	✓	-	✓
**hsa-let-7c-5p**	✓	-	✓
**hsa-miR-193a-5p**	✓	✓	✓
**hsa-miR-885-5p**	-	-	✓
**hsa-miR-376c-3p**	-	-	✓
**hsa-miR-374a-5p**	✓	✓	✓
**hsa-miR-30e-3p**	✓	-	✓

**Table 4:** Differentially expressed miRNA in RP patients versus CD patients with PFS *≥* 8. Fold change (FC) in the rapid progression group (*n* = 18) versus controlled disease group (*n* = 14). The statistical significance of the data was examined using two-tailed Student’s *t* test (*p*-value).

ID miRNA	FC	*p*-Value
hsa-miR-26a-5p	−1.825	0.033
hsa-miR-26b-5p	−1.769	0.018
hsa-miR-485-3p	−2.691	0.013
hsa-miR-126-5p	−1.695	0.027
hsa-miR-23b-3p	−1.524	0.035
hsa-let-7d-5p	−1.628	0.038
hsa-miR-132-3p	−2.332	0.050
hsa-miR-205-5p	−4.766	0.046
hsa-miR-378a-3p	−1.881	0.045
hsa-miR-193a-5p	−2.517	0.042
hsa-miR-885-5p	−3.818	0.041
hsa-miR-2110	−3.771	0.048
hsa-miR-376c-3p	−2.772	0.050
hsa-miR-30e-3p	−2.241	0.012

**Table 5:** Differentially expressed miRNA in RP patients versus CD patients with PFS > 10. Fold change (FC) in the rapid progression group (*n* = 18) versus controlled disease group (*n* = 7). The statistical significance of the data was examined using two-tailed Student’s *t* test (*p*-value).

ID miRNAs	FC RP vs. CD PFS > 10	*p*-Value
hsa-miR-26a-5p	−2.011	0.044
hsa-miR-26b-5p	−1.669	0.044
hsa-miR-485-3p	−3.131	0.022
hsa-miR-339-3p	−2.023	0.011
hsa-miR-382-5p	−3.890	0.034
hsa-miR-106b-5p	−1.620	0.050
hsa-miR-132-3p	−2.730	0.050
hsa-miR-421	2.130	0.046
hsa-miR-205-5p	−3.992	0.035
hsa-miR-378a-3p	−2.353	0.011
hsa-miR-885-5p	−5.349	0.037
hsa-miR-140-5p	−2.376	0.050
hsa-miR-376c-3p	−3.097	0.050
hsa-miR-199a-5p	−3.817	0.041
hsa-miR-363-3p	−1.580	0.043
hsa-miR-30e-3p	−2.561	0.007

**Table 6:** Variation in the circulating miRNA levels in CD patients with PFS > 10 (*n* = 7).

Parameters *	Cycles					*p* ^^^
	1 (T0)	2 (T3)	3 (T6)	4 (T12)	6 (Tpr)	
miR-23b-3p	1.17 (0.92)	1.41 (1.93)	0.69 (0.45)	0.34 (0.33)	0.39 (0.30)	0.001
miR-30e-3p	0.05 (0.13)	0.07 (0.11)	0.02 (0.003)	0.01 (0.03)	0.01 (0.04)	0.02
miR-205-5p	0.19 (0.58)	0.04 (0.04)	0.01 (0.01)	0.01 (0.06)	0.02 (0.24)	0.04

* The mean and standard deviation (M ± SD) for continuous variables. ^ Friedman test. Abbreviation: CD, controlled disease.

## Data Availability

The gastric cancer patients dataset is available on FigShare DOI: 10.6084/m9.figshare.27926331.

## Supplementary Materials

The following supporting information can be downloaded at: https://www.mdpi.com/article/10.3390/ijms252413498/s1.
