# Supplementary figures and images for: Circulating miR-23b-3p, miR-30e-3p, and miR-205-5p as Novel Predictive Biomarkers for Ramucirumab–Paclitaxel Therapy Outcomes in Advanced Gastric Cancer
Source: Int J Mol Sci. 2024 Dec 17;25(24):13498. doi: 10.3390/ijms252413498 (PMC11677161; doi:10.3390/ijms252413498)

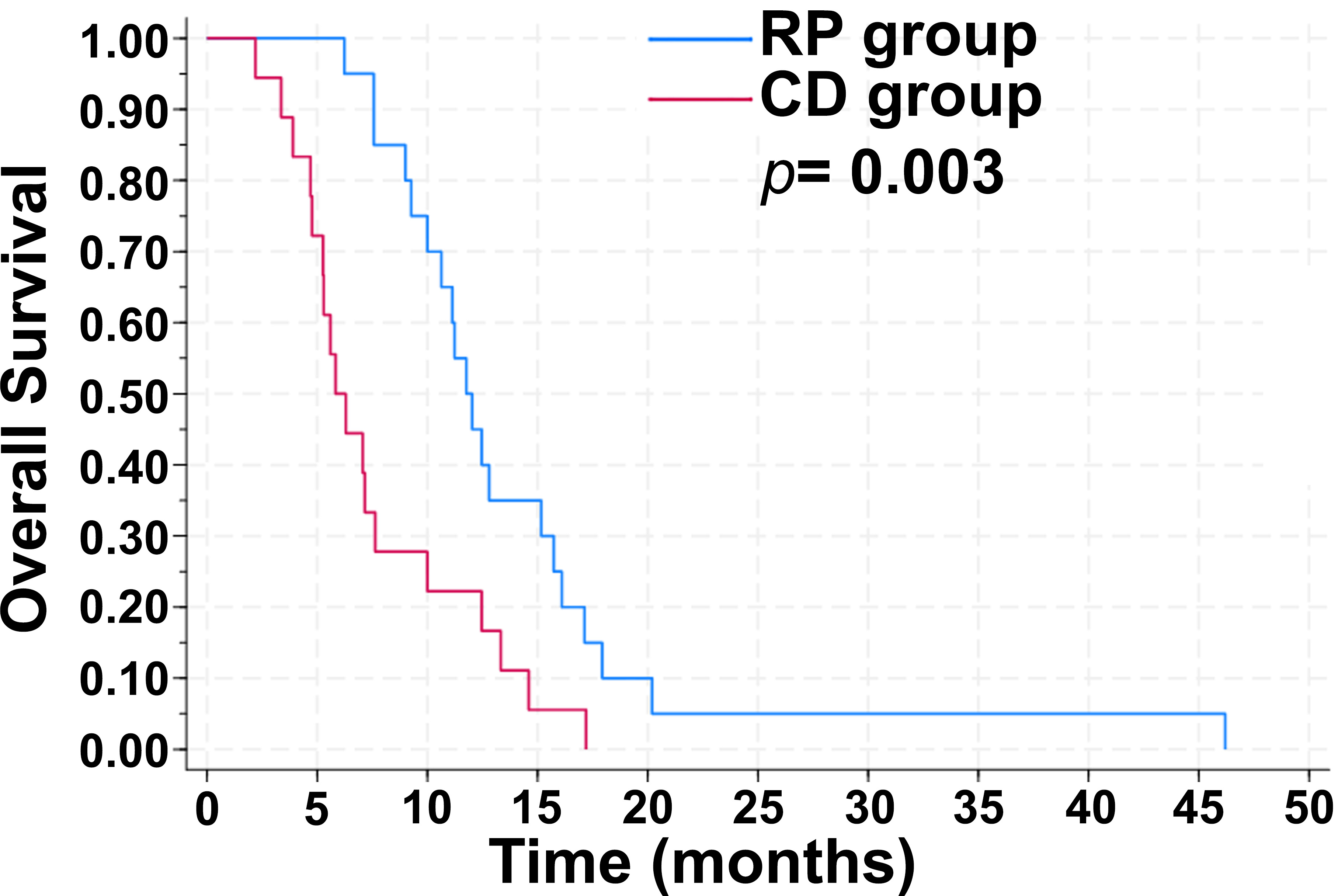

Supplement: Supplementary file 1 [file ijms-25-13498-s001.zip › Figure S1.jpg]
